# Triple Surgical Fixation Technique for an Isolated Greater Trochanter Fracture in an Amateur Weightlifter

**DOI:** 10.5435/JAAOSGlobal-D-21-00127

**Published:** 2022-05-09

**Authors:** Brent Sanderson, Frederic Washburn, Daniel Allison

**Affiliations:** From the Department of Orthopedic Surgery, Community Memorial Health System, Ventura, CA (Dr. Sanderson, and Dr. Washburn); the Cedars-Sinai Medical Center (Dr. Allison); University of Southern California and L.A. County (Dr. Allison); Cedars-Sinai Division of Orthopedic Oncology (Dr. Allison); Children's Hospital of Los Angeles (Dr. Allison); and Medical Corps, United States Navy Reserve (Dr. Allison).

## Abstract

Isolated greater trochanter fractures have been infrequently described in the literature and are typically managed conservatively. Functional strength after injury to the abductor complex can be markedly affected resulting in a Trendelenburg gait and overall abductor weakness. We present a case of a 35-year-old athlete who underwent surgical fixation because of notable fracture displacement and function debility. This case vignette demonstrates the importance of using all available interdisciplinary orthopaedic surgery literature to provide a patient-specific surgical construct. Our patient benefitted from arthroscopic, arthroplasty, and trauma evidence-based medicine to successfully treat his displaced greater trochanteric hip fracture. Successful surgical fixation was enhanced by combining three different methods of fixation: osteosynthesis with partially threaded screws and washers (DePuy Synthes), suture anchor (Arthrex) direct fracture approximation and tendon reinforcement, and a knotless double-row suture bridge (Arthrex) tension band construct. The patient was able to return weightlifting at 4 months postoperatively with no evidence of weakness or trendelenburg gait.

Isolated greater trochanter (GT) fractures are rare injuries, and recent literature has mainly focused on the incidence and treatment of intertrochanteric fracture extension in the elderly and periprosthetic fractures.^1,2,3,4,5^ Magnetic resonance imaging (MRI) is recommended to fully evaluate the extent of the injury and identify notable intertrochanteric extension.^1,4,6,7,8,9^

Surgical indications for isolated greater trochanteric fractures without notable intertrochanteric extension are not clearly defined because of the lack of evidence on the topic.^4,5,6,7^ Young, active patients with large, displaced GT fragments may benefit from surgical fixation as opposed to nonsurgical treatment due to the risk of diminished abductor function.^10^

In this clinical vignette, we describe the clinical course and novel triple fixation surgical technique of a young, active patient who sustained an isolated displaced GT fracture after a motor vehicle collision.

The patient was informed that data concerning the case would be submitted for publication, and he provided consent.

## Case Report

**Figure 1:**
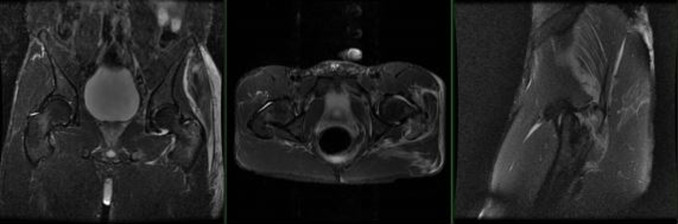
1.5 Tesla MRI of the pelvis demonstrating (coronal, axial, and sagittal slices) an isolated greater trochanteric left hip fracture with minimal displacement and no intertrochanteric extension. Associated left gluteus medius tendinosis without avulsion of the tendinous insertion on the greater trochanter fragment. Increase fluid intensity throughout the gluteus medius and maximus muscle bellies.

**Figure 2:**
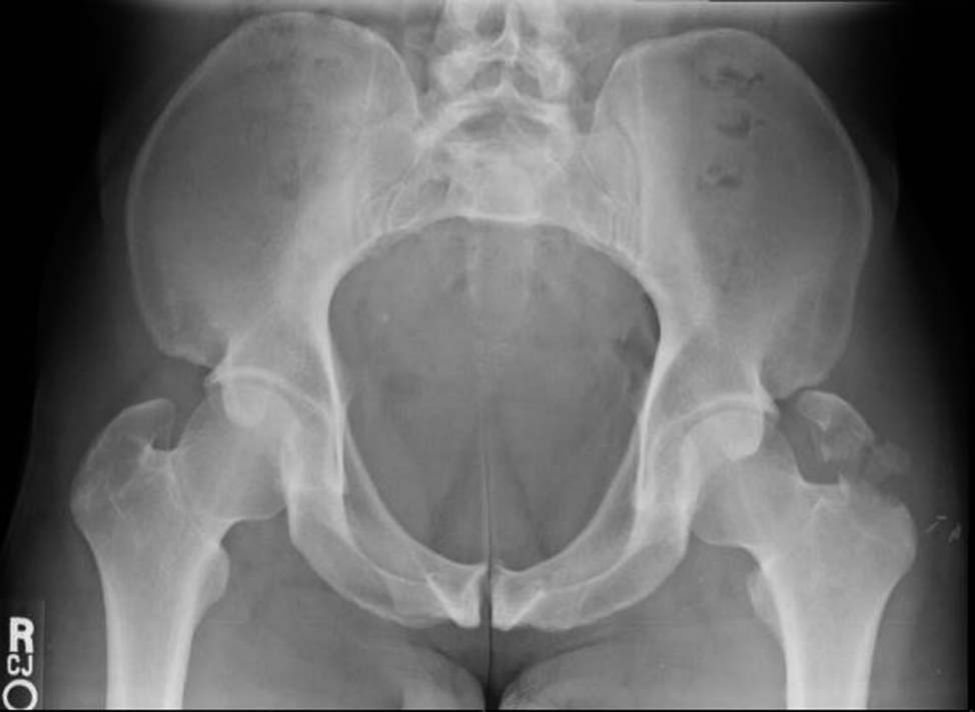
AP pelvis inlet view demonstrating a displaced left greater trochanter fracture with two distinct fracture fragments.

A 35-year-old highly active weightlifter with no medical history presented to the orthopaedic clinic with moderate left lateral hip pain worse with active hip abduction after being involved in a motor vehicle collision. Pain over the lateral hip also occurred during single-leg weight bearing. Radiographs and MRI demonstrated a nondisplaced left GT fracture with gluteus medius tendinosis and no intertrochanteric extension (Figure 1). He was initially treated nonoperatively with touch-down weight bearing as tolerated and strict hip abduction and adduction precautions. However, repeat radiographs completed 6 days after his injury demonstrated notable left GT fracture fragment displacement (Figure 2). His physical examination demonstrated a Trendelenburg gait and focal weakness of the left hip abductors. Treatment options were presented to the patient, which included continued nonsurgical treatment versus surgical intervention. The patient wished to pursue surgical treatment, with the goal of returning to full preinjury function.

## Surgical Technique

A direct lateral approach to the GT was done. Two distinct trochanteric fracture fragments, anteromedial and posterolateral, were identified and cleared of fracture hematoma.

**Figure 3:**
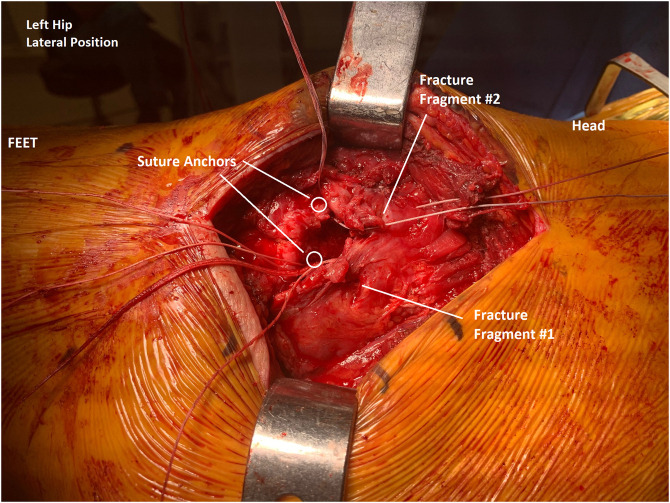
Clinical photograph showing the left hip status after completion of two suture anchor placement (circles). The first two suture tails were placed proximal and slightly anterior to the anterior fracture fragment labeled “Fracture Fragment #2.” Remaining suture tails can be seen distally in preparation of sequential passing through the proximal bone tendon junction.

First, two double-loaded 4.75-mm suture anchors (Arthrex) were placed in each fracture fragment distal trochanteric bed. Pairs of FiberWire suture from the anchors were passed in a horizontal mattress fashion through the bone gluteus medius tendon interface using a free needle, marching from anterior to posterior, ensuring even spacing of the eight suture limbs. The passed suture limbs were placed to the side and left untied (Figure 3).

Second, the fracture was then reduced anatomically and provisionally stabilized with two bone holding tenaculums. Two guidewires were then passed across the fracture site, one in each fragment. Using two cannulated compression screws (DePuy Synthes, Wes), compressive fixation was achieved through each fracture fragment, avoiding the previously placed suture anchors and suture tails. The screws were advanced until the washers were just visible above the GT soft tissue. TigerTape cerclage with four suture tails (TigerTape; Arthrex) was then passed around the screw heads and under the corresponding washers. After ensuring even suture tails, the screws were then tightened sequentially completing our osteosynthesis fixation while maintaining the four free TigerTape suture tails distally. The previously passed FiberWire sutures were then tied over the bony bridge completing the horizontal mattress eight-stranded suture repair. Fluoroscopy and clinically we observed adequate compression at the fracture site.

**Figure 4:**
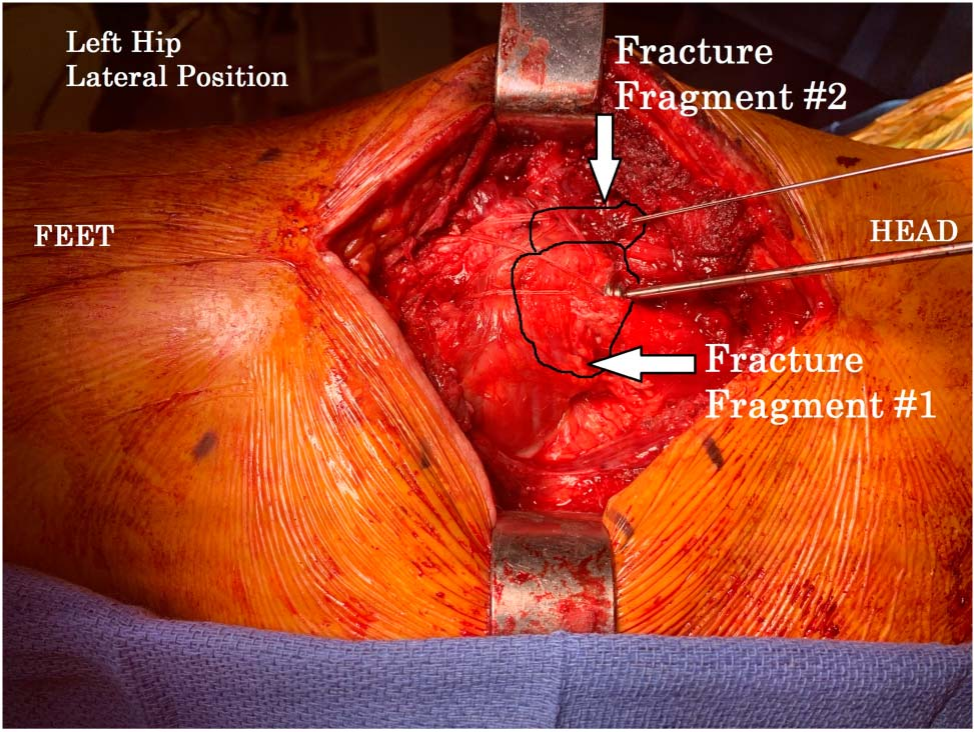
Clinical photograph showing the left hip status after completion of our three fracture fixation constructs before removal of the cannulated screw guidewires. Guidewires remain in place to demonstrate the direction of the underlying compression screws. The double-row suture tape design can be visualized distal to the screw heads.

**Figure 5:**
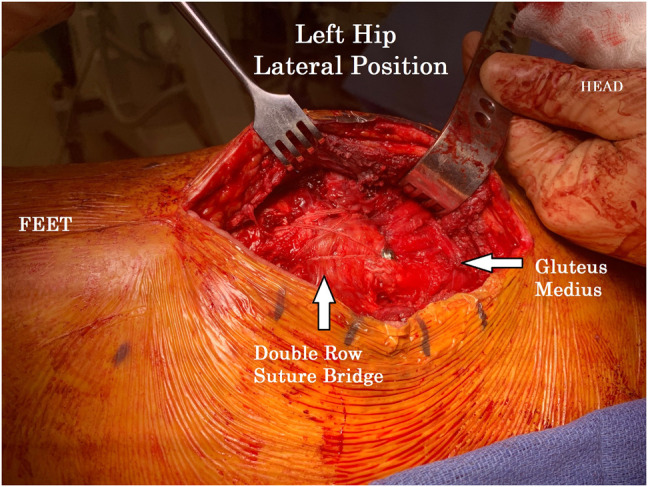
Clinical photograph showing the left hip status after completion of our three fracture fixation constructs. The double-row suture tape design using distal knotless suture anchors and screw heads with washers completed our tension band construct.

**Figure 6:**
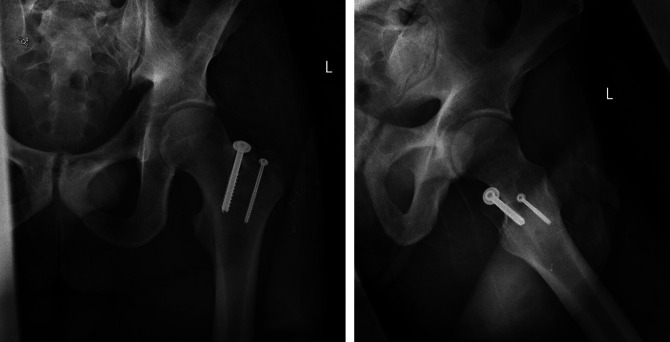
AP and lateral radiographs of the left hip demonstrating anatomic reduction of the left greater trochanteric fracture with stable parallel implant.

Next, the anterior third and posterior thirds of the lateral femur were cleared for insertion of two 4.75-mm biocomposite knotless suture anchors (SwiveLock; Arthrex). One anteromedial TigerTape suture tail and one posterolateral TigerTape tail were then loaded within a 4.75-mm biocomposite knotless suture anchor. The anchor was then reduced to the guide hole in the posterior third of the femur (Figures 4 and 5). This was repeated for the anterior suture anchor incorporating the remaining TigerTape tails. The posterior and anterior anchors were screwed into place while appropriate tension was applied. This completed our knotless double-row suture tape tension band construct. Anatomic reduction and appropriate stable implant placement were again confirmed by multiplanar visualization and orthogonal fluoroscopic imaging (Figure 6).

**Figure 7:**
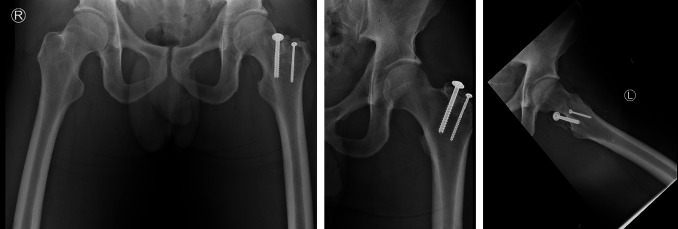
AP pelvis, AP left hip, and lateral radiograph of the left hip demonstrating interval left greater trochanteric fracture heading with stable parallel implant.

Immediate protected weight bearing as tolerated was started on postoperative day zero with crutches provided as needed. Strict hip active abduction and passive adduction precautions were in place for 6 weeks postoperatively. Passive and active hip flexion and extension were started immediately. Progressive hip strengthening began 8 weeks postoperatively with focus on full hip range of motion and proper gait mechanics. The patient returned to weightlifting 4 months postoperatively without symptoms. At the 6-month postoperative visit, strength and range of motion returned to contralateral hip levels with no clinical left hip abductor atrophy. As a weightlifter, his previous recorded maximum left hip abduction was 230 pounds preoperatively, and he returned to this value at 6 months after surgery. Subjectively, the patient was very satisfied with the outcome at the 2-year follow-up with retained full strength and no complaints. Follow-up radiographs demonstrated appropriate fracture healing and stable implant (Figure 7).

## Discussion

The natural history of isolated GT fractures is difficult to generalize, given its rarity and the literature heterogeneity describing clinical outcomes.^2,3,5,6^ MRI is currently the recommended imaging study that reliably demonstrates the actual complexity of these injuries because both CT and bone scan were imprecise in previous studies.^7,9,11,12^ Once the MRI scan has demonstrated no notable intertrochanteric extension, risks and benefits of surgical versus nonsurgical management are to be considered.

To aid in the treatment decision, the arthroplasty literature provides indications for surgical management and technique. Perioperative periprosthetic GT fractures have an incidence as high as 5% in patients undergoing total hip arthroplasty.^13^ However, overall, it is not believed to have a major effect on overall clinical outcome.^2,5,14^ Controversy remains for surgical and nonsurgical indications for GT fractures about a hip prosthesis. Nondisplaced or minimally displaced GT fractures have been historically treated nonoperatively, with activity restriction and restricted weight bearing without need for bracing treatment.^2^ In a recent study by Hartford et al,^5^ all postoperative GT fractures after direct anterior approach hip arthroplasty healed with nonsurgical treatment included protective weight bearing and discontinuation of hip abduction and flexion exercises. This treatment protocol was successful for fractures that were displaced up to 1.5 cm.

Notable displacement of the GT fracture fragment may be limited because of the preservation of the tensor fascia lata and gluteus maximus; however, when the GT fragment undergoes displacement, surgical fixation may be warranted.^2,5,15^ In a retrospective review by Pritchett et al,^2^ they reviewed 30 patients who sustained a fracture of the GT after total hip arthroplasty. When the GT fracture was displaced less than 1 cm, the patients remained asymptomatic with no functional limitations. When the fracture was displaced 1 to 2 cm or more than 2 cm, only 42% or 38% of the patients remained asymptomatic, respectively. By contrast, 63% of the patients with displacement greater than 2 cm became symptomatic. An additional finding was only three patients (10%) had an increase in the amount of fracture displacement more than 2 months after injury. They concluded that isolated fractures of the GT tended to be stable and additional displacement usually does not occur after 2 months. Indications for surgical treatment of GT fractures after total hip arthroplasty, proposed by Pritchett,^2^ were dislocation or instability of the prosthesis, severe limp or pain, and widely displaced trochanter fracture >2 cm. In our patient, the radiographs showed more than 2 cm of fracture displacement.

Greater trochanteric osteotomies and periprosthetic GT fractures provide important insight into treatment strategies. The cerclage systems for fixation of greater trochanteric osteotomies progressed from wires to cables to cable grip and plate systems. The high reported union rates with cables, cable grip systems, and locking plates were combined with complications from metal debris generation and implant irritation.^3^ Biomechanical studies found that the cable grip system provided the strongest fixation and results in low rates of nonunion and trochanteric migration.^16^ An important finding in clinical studies was that a notable improvement in function was only achieved with the osseous union. These findings provide evidence in favor of surgical treatment with the goal of anatomic GT fracture reduction.^17^ Nonunion has been proposed to result in pain in the trochanteric region, functional gait abnormality, and reduced hip abductor strength.^10,17^

Although robust-specific biomechanical and clinical research regarding fixation for native isolated GT fractures is lacking, evidence from various tendon repair techniques and sports literature can be beneficial during surgical planning. Surgical treatment of gluteus medius tendinous injuries at the GT anatomical insertion site has been well described with a variety of open and endoscopic options. DeFroda et al^18^ reported on 31 patients undergoing mini-open gluteus medius repair with double-row suture anchor fixation. The short-term results from their study showed statistically significant improvements in postoperative hip range of motion and superior patient-reported outcomes at 6 months. Furthermore, a recent hamstring tendon biomechanical study by Gerhardt et al^19^ found that an all-knotless suture anchor repair construct resulted in the highest maximal load to failure. A double-row all-knotless repair provides increased strength and ultimately increased healing potential without relying on the variability of knot tying.^20^

Our final construct made use of three evidence-based fixation modalities: osteosynthesis with partially threaded screws through the two fracture fragments, suture anchor direct tendon reinforcement, and a double-row all-knotless suture bridge tension band construct. The tension band construct will act by transmitting the active hip abduction force into a compressive force at the fracture site. The ultimate goal in our patient was to provide strong fracture fixation, through biomechanical and clinically proven surgical techniques, allowing for early physical therapy and limited surgical site morbidity. This triple fixation technique may be used in cases with multiple fracture fragments and in patients seeking to shorten their recovery time. However, the benefits of decreased recovery time must be weighed against the increased surgical time and implant cost. Thus, the triple fixation technique may not apply to all patients with an isolated GT fracture. This case demonstrates the importance of evidence-based surgical technique preoperative planning and patient-specific fixation strategies when treating uncommon fractures.
